# Comprehensive angiographic evaluation of graft quality after endoscopic vein harvesting in coronary artery bypass grafting

**DOI:** 10.3389/fcvm.2026.1767383

**Published:** 2026-02-03

**Authors:** Ken Nakamura, Kentaro Akabane, Shusuke Arai, Ryota Katsura, Miku Konaka, Jun Hayashi, Eiichi Ohba, Cholsu Kim, Hideaki Uchino, Takao Shimanuki, Tetsuro Uchida

**Affiliations:** 1 Division of Cardiovascular Surgery, Nihonkai General Hospital, Sakata, Japan; 2 Second Department of Surgery, Yamagata University Faculty of Medicine, Yamagata, Japan

**Keywords:** coronary angiogram, coronary artery bypass grafting, endoscopic vein harvest, graft patency, saphenous vein graft

## Abstract

**Background:**

The saphenous vein graft (SVG) remains a mainstay conduit for coronary artery bypass grafting (CABG) due to its accessibility and length. Although the no-touch technique may improve long-term patency, wound complications are a continuing concern. Since 2011, our institution has adopted endoscopic vein harvesting (EVH) as the standard approach. This study provides angiographic insights into graft quality and patency after EVH compared with open vein harvesting (OVH), with additional assessment of mid-term clinical outcomes.

**Methods:**

Among 471 patients who underwent CABG between 2005 and 2017, 307 were included in this study. Patients were divided into the EVH group (Group A, *n* = 134) and the OVH group (Group B, *n* = 173). Postoperative coronary angiography was used to evaluate SVG graft patency, anastomotic integrity, and graft body stenosis. Clinical outcomes including major adverse cardiac and cerebrovascular events (MACCE) and wound complications were also compared.

**Results:**

Angiographic assessment demonstrated comparable SVG patency between the EVH and OVH groups (93% vs. 94%), with similar rates of anastomotic stenosis (2.2% vs. 2.3%) and severe graft stenosis (≥90%; 1.5% vs. 1.2%). No significant differences were observed in 30-day mortality (1.5% vs. 3.5%), in-hospital mortality (1.5% vs. 2.1%), or postoperative stroke. Wound-related complications were rare, including wound dehiscence (1.5% vs. 2.3%) and infection (0.7% vs. 1.2%). MACCE-free survival rates at 1, 3, and 5 years were 97%, 94%, and 91% in the EVH group vs. 92%, 86%, and 76% in the OVH group, respectively (*p* = 0.070), showing a favorable trend in the EVH group.

**Conclusion:**

Detailed angiographic evaluation revealed that EVH did not compromise graft quality or patency compared with conventional OVH. The incidence of wound complications was very low, and early postoperative SVG-related events were favorable. These findings suggest that EVH is a safe and reliable harvesting technique, providing high-quality grafts with excellent angiographic integrity. Individualized selection of harvesting strategy remains important for optimizing surgical outcomes.

## Introduction

The great saphenous vein (SVG) remains the most widely used second conduit in coronary artery bypass grafting (CABG) after the internal thoracic artery (ITA), owing to its adequate length and relatively low risk of vessel injury during harvesting (1, 2). While historically associated with inferior long-term patency compared to arterial grafts, recent studies have reported favorable long-term outcomes with SVGs (3). The no-touch harvesting technique, which avoids direct manipulation of the vein, has been proposed to improve graft durability and patency (4, 5); however, recent randomized controlled trials, such as the SWEDEGRAFT trial, have shown no significant difference in vein graft failure between no-touch and conventional harvesting methods, while highlighting a higher incidence of wound complications associated with the no-touch approach (6). Endoscopic vein harvesting (EVH) has emerged as a widely adopted technique, particularly in the United States and Europe, offering significant advantages in terms of reduced wound morbidity and faster recovery (7, 8). Unlike the no-touch method, EVH provides a minimally invasive alternative that prioritizes patient recovery and wound care. Previous studies have primarily evaluated EVH outcomes based on clinical events or overall graft patency rates, without detailed angiographic assessment of specific lesion sites. Consequently, the relationship between the harvesting technique and localized graft abnormalities—such as anastomotic narrowing, graft body stenosis, or distal runoff compromise—remains insufficiently understood.

In this study, we aimed to provide angiographic insights into graft quality and patency following EVH, directly comparing it with conventional open vein harvesting (OVH). By integrating detailed postoperative coronary angiography (CAG) findings with early postoperative SVG-related events, we sought to clarify whether EVH compromises graft integrity or durability. This comprehensive imaging-based evaluation offers a novel perspective on the safety and effectiveness of EVH in contemporary CABG practice.

## Patients and methods

This was a retrospective observational study conducted at two centers, Nihonkai General Hospital. A total of 471 consecutive patients who underwent isolated or combined CABG between December 2005 and December 2017 were included. Data were accessed for research purposes between 01/04/2015 and 31/12/2019. The study protocol was approved by the Institutional Review Board of Nihonkai General Hospital (Approval No. 007-4-12). Owing to the retrospective study design, the requirement for additional written informed consent was waived, although all patients had provided appropriate informed consent for treatment and data use at the time of care. The research was performed in compliance with the principles of the Declaration of Helsinki.

This study aimed to compare graft patency and stenosis rates between SVGs harvested using EVH and those obtained via conventional open harvesting techniques. In addition, we assessed long-term graft patency and the incidence of MACCE.

Eligible patients included those who underwent CABG with at least one SVG harvested using the EVH technique. Patients who required intraoperative conversion to open vein harvesting were excluded from the analysis. Postoperative graft assessment was conducted through qualitative evaluation using coronary angiography (CAG). Patients presenting with lower-extremity varicose veins were excluded from consideration for SVG harvesting. To evaluate the suitability of the vein, preoperative CT vein mapping was routinely performed. EVH was conducted simultaneously with the harvesting of the left internal mammary artery by an assistant surgeon, utilizing a standardized technique (VirtuoSaph system; Terumo Cardiovascular, Ann Arbor, MI). The EVH procedure—comprising skin incision, blunt tissue dissection, branch ligation, and graft preparation—was performed in accordance with established protocols previously reported in the literature (9).

The primary endpoint of this study was the incidence of early graft-related complications, such as occlusion, stenosis, or wound infection. Secondary endpoints included long-term graft patency and MACCE, defined as a composite of death, myocardial infarction, stroke, or any revascularization procedure, including coronary or non-coronary interventions (e.g., peripheral or carotid artery revascularization). Postoperative atrial fibrillation was diagnosed based on documented episodes lasting more than 30 s during hospitalization. Neurological events were included only when supported by findings on CT or MRI and confirmed by a neurosurgical consultation; cases suggestive of TIA without imaging evidence were excluded.

Preoperative optimization involved the management of comorbidities such as dental infections, uncontrolled diabetes mellitus, and carotid artery stenosis. All patients participated in a structured perioperative rehabilitation program under the supervision of physical therapists.

The choice between on-pump and off-pump CABG (OPCAB) was made during a multidisciplinary preoperative conference, taking into account the patient's anatomical and clinical characteristics. On-pump CABG was preferred in cases with ventricular enlargement, reduced cardiac function, or anatomically complex targets. OPCAB was selected when complete revascularization appeared feasible on a beating heart. Conversion to cardiopulmonary bypass (CPB) was initiated intraoperatively in response to hemodynamic compromise, including ventricular arrhythmias, hypotension (systolic blood pressure ≤80 mmHg), or cardiac arrest. In the institutions included in this study, all coronary anastomoses were performed exclusively by experienced cardiac surgeons with more than five years of independent open heart surgery experience. While the experience level of vein harvesters varied, no novice surgeons were involved in the performance of coronary anastomosis. In addition, all harvested vein grafts were routinely inspected and quality-checked by the operating surgeon prior to anastomosis.

During OPCAB, cardiac exposure and stabilization were achieved using posterior pericardial stay sutures, gauze packing, a tissue stabilizer (Octopus; Medtronic, Minneapolis, MN), positional adjustments, and adjuncts such as CO₂ insufflation and saline misting as needed. In on-pump CABG, the same grafting strategy was followed, with beating-heart techniques employed whenever feasible. Prophylactic intra-aortic balloon pump (IABP) support was administered preoperatively to selected high-risk patients, in accordance with indications previously reported by our institution (10).

In the vast majority of cases, the left internal mammary artery was anastomosed to the left anterior descending artery. Additional revascularization of the circumflex and right coronary territories was performed using radial artery grafts or saphenous vein grafts (SVGs). A no-touch aortic technique employing bilateral internal mammary arteries was utilized in patients with suspected ascending aortic calcification or sclerosis based on imaging or intraoperative palpation. Graft quality was routinely assessed intraoperatively using a transit-time flow measurement device (Butterfly Flowmeter; Medistim, Oslo, Norway).

### Statistical analysis

Continuous variables were presented as either mean ± standard deviation or median with interquartile range, depending on data distribution. Categorical variables were summarized as counts and percentages. For comparisons of continuous variables, either the independent Student's *t*-test or the Mann–Whitney *U*-test was applied, as appropriate. Categorical data were evaluated using the chi-square test or Fisher's exact test, as indicated.

Kaplan–Meier survival analysis was used to estimate MACCE-free survival and freedom from SVG-related events in both groups, with comparisons made using the log-rank test. All statistical analyses were performed using JMP software, version 18.2.0 (SAS Institute Japan, Tokyo, Japan).

## Results

**Figure 1:**
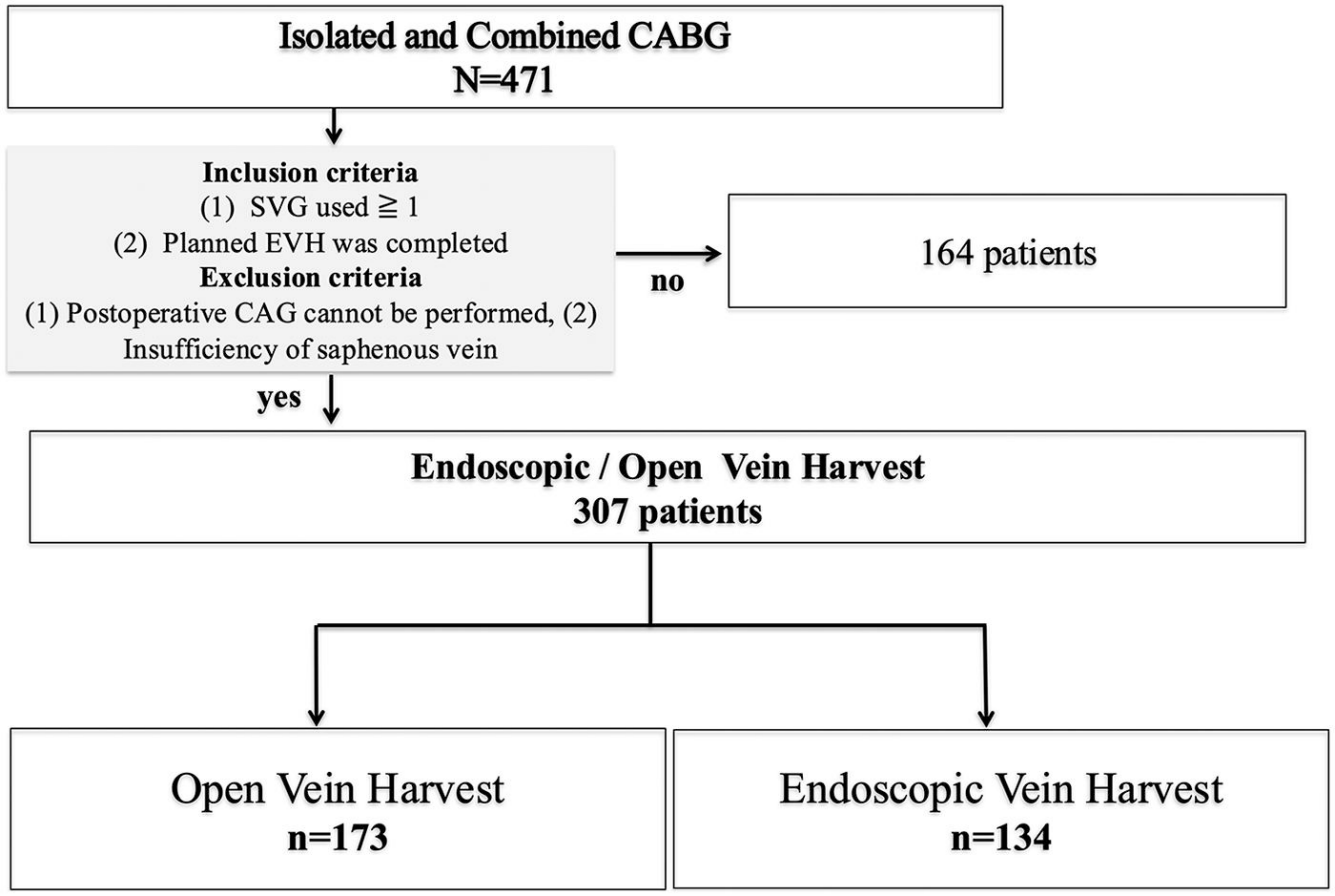
Patient selection and grouping based on vein harvesting technique. Among 471 patients who underwent isolated or combined coronary artery bypass grafting (CABG), a total of 307 patients met the inclusion criteria—use of ≥1 saphenous vein graft (SVG) and successful completion of planned endoscopic vein harvesting (EVH)—and did not meet the exclusion criteria—postoperative coronary angiography (CAG) not feasible or insufficient saphenous vein quality. These 307 patients were categorized into two groups based on the vein harvesting technique: 134 patients in the EVH group and 173 patients in the open vein harvesting (OVH) group. Comparative analyses were conducted between these two cohorts. CABG, coronary-artery bypass grafting; SVG, saphenous-vein graft; EVH, endoscopic vein harvesting; CAG, coronary angiography; OVH, open vein harvesting.

A total of 471 consecutive patients were included in this study. Among them, 134 underwent EVH and 173 underwent OVH, as shown in Figure 1. Preoperative clinical characteristics are summarized in Table 1. There were no significant differences between the two groups in age, sex, BMI, or comorbidities such as hypertension and diabetes mellitus. However, the incidence of prior myocardial infarction was significantly lower in the EVH group compared to the OVH group (47% vs. 62%, *p* < 0.05). The mean follow-up duration was 21 ± 18 months in the EVH group and 47 ± 45 months in the OVH group (*p* < 0.0001), which likely reflects the temporal introduction of EVH beginning in 2011 at our institution. Aside from this time-related difference, baseline characteristics were largely comparable between the groups. EuroSCORE II was 2.5 ± 3.1 vs. 2.5 ± 3.0 (*p* = 0.982), and LVEF was 53 ± 15% vs. 54 ± 16% (*p* = 0.763), respectively (Table 1).

**Table 1:** Baseline patient characteristics (preoperative data).

Characteristic	Open vein harvest (*n* = 173)	Endoscopic vein harvest (*n* = 134)	*p*-value
Age, years	69 ± 10	69 ± 9	0.821
Male sex, *n* (%)	138 (81)	114 (84)	0.451
BMI, kg/m²	23 ± 4	24 ± 4	0.308
BMI ≥30, *n* (%)	10 (6)	5 (4)	0.433
Risk factors, *n* (%)			
Hypertension	135 (80)	108 (80)	1
Hyperlipidemia	113 (68)	89 (66)	0.806
Diabetes mellitus	82 (49)	64 (47)	0.908
Insulin use	24 (14)	11 (8)	0.147
Smoking history	62 (60)	81 (69)	0.202
Current smoker	18 (17)	11 (9)	0.073
Old myocardial infarction, *n* (%)	104 (62)	63 (47)	<0.05
Family history, *n* (%)	21 (24)	17 (16)	0.208
Prior PCI, *n* (%)	37 (22)	19 (14)	0.102
Peripheral arterial disease, *n* (%)	14 (8)	12 (9)	0.839
CKD *n* (%)	22 (13)	14 (10)	0.593
Hemodialysis, *n* (%)	11 (6)	8 (6)	1
Prior stroke, *n* (%)	12 (7)	13 (10)	0.529
NYHA class III–IV, *n* (%)	61 (36)	37 (27)	0.139
Left ventricular ejection fraction, %	54 ± 16	53 ± 15	0.763
Left main coronary artery stenosis, %	57 ± 33	58 ± 43	0.096
Number of diseased vessels ≥50%	2.6 ± 0.6	2.6 ± 0.6	0.746
Emergency/urgent surgery, *n* (%)	20 (12)	15 (11)	1
EuroSCORE II, median (IQR)	1.48 (0.92–2.74)	1.57 (0.95–2.81)	0.982
Follow-up duration, months, median (IQR)	31 (4–86)	17 (4–34)	<0.0001

Continuous variables are presented as mean ± standard deviation.

OVH, open vein harvesting; EVH, endoscopic vein harvesting; BMI, body mass index; PCI, percutaneous coronary intervention; CKD, chronic kidney disease; NYHA, New York heart association, IQR, Interquartile Range.

The number of distal anastomoses was significantly lower in the EVH group compared to the OVH group (2.7 ± 0.9 vs. 3.0 ± 0.9, *p* < 0.005), and fewer SVGs were used in the EVH group (1.3 ± 0.6 vs. 1.5 ± 0.7, *p* < 0.05). The use of LITA was significantly more frequent in the EVH group [126/134 [93%] vs. 147/173 [85%], *p* < 0.05], while the use of other types of grafts did not differ significantly between the two groups.

Early postoperative SVG occlusion occurred in 9 cases (6.7%) in the EVH group and 11 cases (6.4%) in the OVH group, with no significant difference (*p* = 0.551). Similarly, SVG stenosis of less than 50% was observed in 1 case (0.7%) in the EVH group and none in the OVH group (*p* = 0.440). SVG stenosis of ≥90% was comparable between groups [2/134 [1.5%] vs. 2/173 [1.2%], *p* = 0.593], while stenosis of 50%–90% tended to be more frequent in the EVH group, though this did not reach statistical significance [5/134 [3.7%] vs. 1/173 [0.6%], *p* = 0.090]. Importantly, detailed angiographic review revealed no EVH-specific patterns of graft injury. Most graft body stenoses were observed in proximity to venous valves or at sites of graft kinking related to graft routing and configuration, rather than diffuse narrowing suggestive of intrinsic graft wall damage. In these cases, antegrade flow to the target coronary artery was preserved, and re-intervention was not required.

Stenosis at the SVG anastomosis site was identified in 3 cases (2.2%) in the EVH group and 4 cases (2.3%) in the OVH group (*p* = 0.665). Re-intervention for SVG stenosis was performed in 2 cases (1.5%) in the EVH group and 3 cases (1.7%) in the OVH group, with no significant difference (*p* = 0.730). Anastomotic stenosis was predominantly associated with unfavorable native coronary anatomy, including small-caliber vessels, poor distal runoff, or the need for intraluminal shunting or bougie dilation during anastomosis. No cases were judged to have anastomotic stenosis attributable to graft fragility or structural deterioration related to the vein harvesting technique. Wound dehiscence was reported in 2 patients (1.5%) in the EVH group (*p* = 0.698), and only 1 of these cases (0.7%) was associated with wound infection (Table 2).

**Table 2:** Assessment of bypass graft anastomosis and wound complication.

Characteristic	Open vein harvest (*n* = 173)	Endoscopic vein harvest (*n* = 134)	*p*-value
Distal anastomoses, *n*, Mean ± SD	3.0 ± 0.9	2.7 ± 0.9	*<0*.*005*
Distal anastomoses SVG used, *n*, Mean ± SD	1.5 ± 0.7	1.3 ± 0.6	*<0*.*05*
Graft selection			
LITA, %	147 (85%)	126 (93%)	*<0*.*05*
RITA, %	4 (2%)	3 (2%)	*1*.*000*
RA, %	56 (33%)	53 (39%)	*0*.*232*
GEA, %	4 (2%)	0 (0%)	*0*.*134*
Early results after SVG harvest			
SVG occlusion early postoperative, %	11 (6.4%)	9 (6.7%)	*0*.*551*
Leg wound problems, %	4 (2.3%)	2 (1.5%)	*0*.*698*
Leg wound infection, %	2 (1.1%)	1 (0.7%)	*0*.*826*
SVG stenosis, <50%, %	0 (0%)	1 (0.7%)	*0*.*440*
SVG stenosis, 50–90%, %	1 (0.6%)	5 (3.7%)	*0*.*090*
SVG stenosis, ≥90%, %	2 (1.2%)	2 (1.5%)	*0*.*593*
Stenosis of SVG anastomosis, %	4 (2.3%)	3 (2.2%)	*0*.*665*
Reintervention to SVG, %	3 (1.7%)	2 (1.5%)	*0*.*730*

Leg wound problems were defined as delayed wound healing or local wound complications at the vein harvest site without clinical or microbiological evidence of infection.

Leg wound infection was defined as the presence of clinical signs of infection (e.g., purulent discharge, erythema, warmth), positive wound culture, and/or systemic findings such as fever or elevated inflammatory markers.

SVG, saphenous vein graft; LITA, left internal thoracic artery; RITA, right internal thoracic artery; RA, radial artery; GEA, gastroepiploic artery.

Italicized values indicate *P* values. *P* values < 0.05 were considered statistically significant.

**Figure 2:**
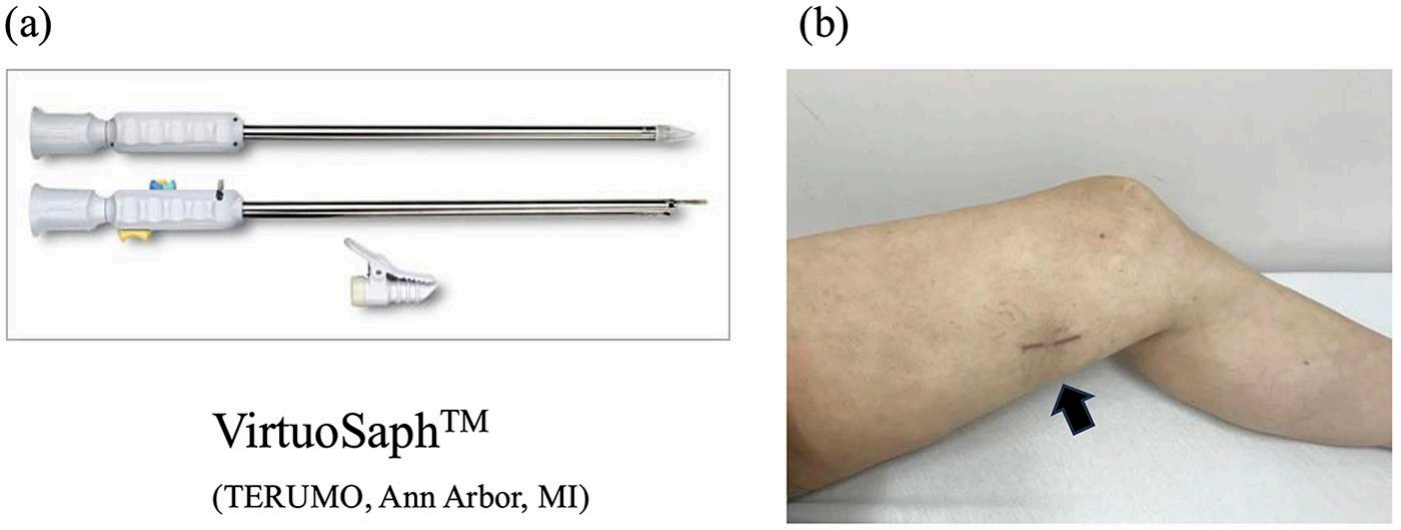
Endoscopic vein harvesting using the virtuoSaph system and postoperative wound appearance. **(a)** The VirtuoSaph system consists of two interchangeable components used sequentially to perform skin incision, blunt tissue dissection, branch ligation, and graft preparation. **(b)** Postoperative view of the distal left thigh incision site. Patients rarely report pain during the postoperative course.

The EVH procedure employed in this study utilized the VirtuoSaph system (Terumo, Ann Arbor, MI), which consists of two separate components designed to independently perform vein dissection and branch ligation (Figure 2a). The skin incision required for this system is minimal and cosmetically favorable, as shown in Figure 2b, and patients rarely reported postoperative wound pain or discomfort at the harvest site.

Intraoperative and postoperative outcomes are summarized in Table 3. No significant differences were observed between the two groups regarding the incidence of combined procedures with CABG, conversion to on-pump CABG, the need for red blood cell transfusion, peak postoperative creatinine levels, occurrence of postoperative atrial fibrillation, mediastinitis, or neurological events. Operative time was significantly shorter in the EVH group than in the OVH group (262 ± 67 min vs. 318 ± 90 min, *p* < 0.0001). The rate of OPCAB was also significantly higher in the EVH group [61/134 [46%] vs. 55/173 [33%], *p* < 0.05]. CPB time tended to be shorter in the EVH group (137 ± 45 min vs. 150 ± 57 min, *p* = 0.065), though the difference was not statistically significant. There were no significant differences in the duration of mechanical ventilation (1.3 ± 1.6 days vs. 1.5 ± 1.9 days, *p* = 0.270) or ICU stay (4.7 ± 3.2 days vs. 5.2 ± 5.7 days, *p* = 0.427). However, postoperative hospital stay was significantly shorter in the EVH group (22 ± 11 days vs. 26 ± 21 days, *p* = 0.019).

**Table 3:** Clinical outcomes and complications according to vein harvesting technique.

Result	Open vein harvest (*n* = 173)	Endoscopic vein harvest (*n* = 134)	*p*-value
Combined CABG, %	25 (15%)	28 (21%)	*0*.*172*
Operation time, min, Mean ± SD	318 ± 90	262 ± 67	*<0*.*0001*
Off-Pump CABG, %	55 (33%)	61 (46%)	*<0*.*05*
Cardiopulmonary bypass, min, Mean ± SD	150 ± 57	137 ± 45	*0*.*065*
Aortic cross-clamp, min, Mean ± SD	21 ± 42	15 ± 30	*0*.*143*
Converted to on-pump CABG, %	3 (5.5%)	3 (4.9%)	*0*.*538*
Required transfusion of red blood cells, %	123 (72%)	93 (69%)	*0*.*614*
Postoperative Max SCr, mg/dL, Mean ± SD	1.9 ± 1.9	2.0 ± 5.2	*0*.*831*
Neurologic dysfunction, %	12 (7%)	13 (10%)	*0*.*529*
Duration of mechanical ventilation (post operative days), median (IQR)	1 (1–1)	1 (1–1)	*0*.*270*
ICU stay (post operative days), Mean ± SD	5.2 ± 5.7	4.7 ± 3.2	*0*.*427*
Length of hospital stay.days, median (IQR)	21 (17–28)	18 (15–24)	*<0*.*05*
Post operative atrial fibrillation, %	21 (13%)	21 (16%)	*0*.*510*
Mediastinitis, %	6 (3.7%)	1 (0.7%)	*0*.*133*
30 days mortatlity, %	6 (3.5%)	2 (1.5%)	*0*.*473*
In-hospital deaths, %	8 (4.7%)	2 (1.5%)	*0*.*194*
MACCE, 12 months, %	36 (21%)	6 (4%)	*<0*.*001*
Cardiac Death, 12 months, %	8 (4.7%)	2 (1.5%)	*0*.*194*

CABG, coronary artery bypass grafting; SD, standard deviation; SCr, serum creatinine; IQR, interquartile range; ICU, intensive care unit; MACCE, major adverse cardiac and cerebrovascular events.

Italicized values indicate *P* values. *P* values < 0.05 were considered statistically significant.

The 30-day mortality was 1.5% in the EVH group and 3.5% in the OVH group (*p* = 0.473), and in-hospital mortality was 1.5% vs. 4.7% (*p* = 0.194). One-year postoperative MACCE occurred significantly less frequently in the EVH group (4% vs. 21%, *p* < 0.001) (Table 3).

**Figure 3:**
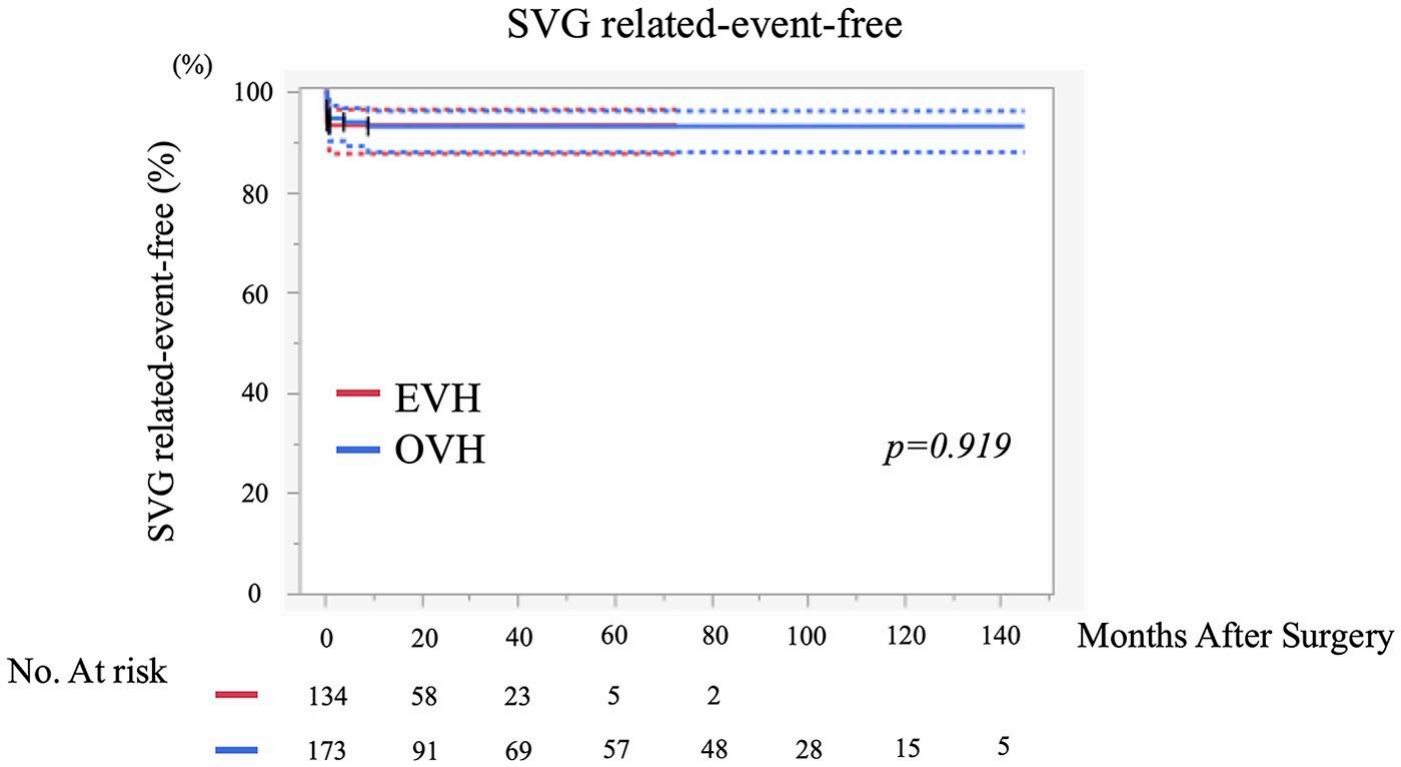
Early saphenous vein graft–related event–free survival according to vein harvesting technique. Kaplan–Meier curves showing early postoperative saphenous vein graft (SVG)–related event–free survival in patients undergoing coronary artery bypass grafting (CABG), stratified by vein harvesting technique. Patients were divided into an endoscopic vein harvesting (EVH) group (*n* = 134) and an open vein harvesting (OVH) group (*n* = 173). Follow-up was continued for up to 140 months, and differences between the groups were evaluated using the log-rank test. SVG, saphenous-vein graft; CABG, coronary-artery bypass grafting; EVH, endoscopic vein harvesting; OVH, open vein harvesting.

**Figure 4:**
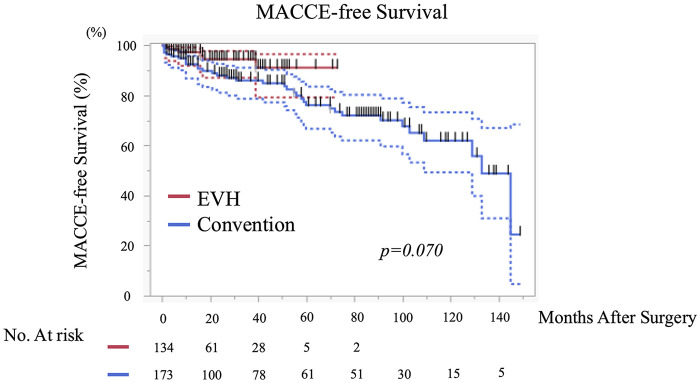
Short- to midterm MACCE-free survival after CABG according to vein harvesting technique. Kaplan–Meier curves depicting short- to midterm major adverse cardiac and cerebrovascular event (MACCE)–free survival following coronary artery bypass grafting (CABG). Patients were stratified according to the saphenous vein harvesting technique: endoscopic vein harvesting (EVH; *n* = 134) and open vein harvesting (OVH; *n* = 173). MACCE was defined as a composite of all-cause death, myocardial infarction, stroke, or any repeat revascularization procedure. Given the retrospective design and the lack of protocol-driven long-term angiographic follow-up, the present analysis primarily reflects early and intermediate clinical outcomes rather than late graft-related events. Comparison between the two groups was performed using the log-rank test. MACCE, major adverse cardiac and cerebrovascular events; CABG, coronary-artery bypass grafting; SVG, saphenous-vein graft; EVH, endoscopic vein harvesting; OVH, open vein harvesting.

Cardiac death occurred in 1.5% of patients in the EVH group and 4.7% in the OVH group (*p* = 0.194). No fatal events were reported after hospital discharge in either group. The SVG-related event-free survival at 1, 3, and 5 years was identical between the groups: 93%/93%/93% in the EVH group and 93%/93%/93% in the OVH group (*p* = 0.919), with no significant differences observed (Figure 3). In the Kaplan–Meier analysis for MACCE-free survival, the event-free rates at 1, 3, and 5 years were 97%, 94%, and 91% in the EVH group, and 92%, 86%, and 76% in the OVH group, respectively (*p* = 0.070), showing a favorable trend in the EVH group (Figure 4).

## Discussion

SVG plays an essential role as the second conduit in CABG following IMA, and its quality may directly influence long-term survival and the incidence of major cardiovascular events. In addition to conventional open vein harvesting, techniques such as the no-touch method—designed to preserve the perivascular tissue—have been associated with improved long-term patency (4, 11). Meanwhile, EVH has gained attention due to its advantages in reducing wound-related complications, such as infection and pain. Recent studies suggest that the method of SVG harvesting may significantly impact both graft patency and clinical outcomes after CABG (12, 13).

The present study yielded several important insights: (1) EVH did not demonstrate any inferiority compared to OVH in terms of graft quality, as evidenced by comparable rates of SVG stenosis and occlusion on follow-up imaging. (2) The incidence of wound-related complications associated with EVH was low, occurring in less than 2%, and no cases of refractory or deep wound infections were observed. (3) There was no indication that EVH had a negative impact on early postoperative SVG-related events in patients undergoing CABG.

These findings are consistent with previous reports suggesting that EVH can maintain graft quality while significantly reducing wound-related complications. Studies such as PREVENT IV and others have previously raised concerns regarding EVH and long-term graft patency (14, 15). However, more recent analyses and meta-analyses have reported that when EVH is performed using a standardized technique, clinical outcomes and graft patency are comparable to those of OVH (16, 17). Our results further support the notion that EVH offers a safe and effective conduit harvesting strategy in contemporary CABG. A distinctive strength of the present study lies in the detailed qualitative assessment of SVGs using early postoperative coronary angiography. Unlike many previous investigations that relied primarily on clinical events or binary patency assessments, we evaluated specific angiographic features such as graft body stenosis and anastomotic site narrowing. This approach allows for a more nuanced assessment of graft quality in the early postoperative phase, during which technical or harvesting-related injury is most likely to manifest.

In a study by J. Ran et al., the incidence of early SVG occlusion was reported as 11.6% in the EVH group and 9.8% in the OVH group, with no significant difference between the two techniques. Similarly, in our study, the rates were 6.7% for EVH and 6.4% for OVH, which are comparable and consistent with these previous findings (18). Another report indicated that graft patency rates between EVH and OVH remained similar up to 6 months postoperatively, with a decline in EVH graft patency observed at 12 months. However, that study did not include a detailed anatomical or morphological evaluation of the grafts, and the reason for the divergence in patency after 12 months remains unclear (19).

While the superiority of EVH over OVH in terms of postoperative wound pain, wound healing, and infection has become widely accepted and is no longer a matter of debate, the underlying mechanisms continue to be explored. Notably, lymphoscintigraphic data have suggested that improved lymphatic drainage in EVH may account for its favorable outcomes, offering a micro-level explanation for the reduced incidence of wound-related complications (20).

In parallel, numerous studies have focused on improving long-term outcomes through the use of no-touch saphenous vein grafts (NT-SVG), based on the hypothesis that preservation of perivascular tissue may enhance graft durability. While early observational studies suggested superior long-term patency with NT-SVG, recent large randomized trials, including SWEDEGRAFT, have demonstrated comparable graft failure rates between no-touch and conventional harvesting, alongside a higher incidence of wound-related complications in the no-touch group (21).

Against this background, EVH and NT-SVG represent fundamentally different strategies with distinct risk–benefit profiles. NT-SVG prioritizes biological preservation of the vein at the expense of increased wound morbidity, whereas EVH emphasizes minimally invasive access and reduced wound complications while relying on meticulous endoscopic technique to preserve graft integrity. Rather than viewing these techniques as competing alternatives, their roles should be contextualized within contemporary evidence and individualized according to patient-specific factors such as frailty, obesity, diabetes, peripheral vascular disease, and expected life expectancy. Given their differing profiles, the selection of an appropriate harvesting method should be tailored to the individual patient's clinical characteristics, which may enhance overall surgical outcomes (6, 12).

First, postoperative coronary angiography was performed predominantly for clinical indications rather than according to a standardized follow-up protocol. As a result, detection bias cannot be excluded, and late graft occlusion or progressive stenosis in asymptomatic patients may not have been adequately captured. Accordingly, the SVG-related event-free survival curves presented in this study predominantly reflect early postoperative outcomes and should be interpreted as indicators of early graft-related safety and wound outcomes rather than definitive assessments of mid- to long-term graft durability.

Second, there was a chronological imbalance between the EVH and OVH groups, as EVH was introduced at our institution in 2011, resulting in a shorter follow-up duration for the EVH cohort. This temporal difference introduces the possibility of bias related to evolving surgical techniques, perioperative management strategies, and institutional experience. Although this reflects real-world clinical practice, such heterogeneity limits direct causal inference regarding the superiority or non-inferiority of EVH.

Third, operative time, cardiopulmonary bypass time, and aortic cross-clamp time may have been influenced by unmeasured procedural complexity, including concomitant surgical procedures performed in addition to CABG. Because the primary focus of this study was the impact of vein harvesting techniques rather than detailed stratification of operative complexity, these factors were not fully adjusted for and should be interpreted with caution. This limitation may reduce the clinical interpretability of between-group differences in operative time-related variables.

Fourth, the retrospective, non-randomized design and relatively small number of angiographically confirmed events limited the statistical power to detect differences in rare outcomes such as severe graft stenosis or reintervention. For this reason, we intentionally did not apply formal adjustment methods such as propensity score matching or inverse probability of treatment weighting, as these approaches could have resulted in model instability and overfitting. While baseline characteristics were largely comparable, residual confounding due to unmeasured variables cannot be excluded, and the findings should therefore be interpreted as hypothesis-generating rather than confirmatory.

Finally, this study was conducted at a limited number of institutions, which may restrict the generalizability of the results to other centers or international practice patterns. Future multicenter prospective studies incorporating standardized angiographic follow-up protocols, longer observation periods, appropriate adjustment strategies, and formal cost-effectiveness analyses are warranted to more comprehensively define the long-term clinical and economic value of EVH in contemporary CABG practice.

Within the context of these limitations, the present study supports the procedural safety and short- to midterm clinical feasibility of EVH, rather than establishing definitive long-term equivalence with open vein harvesting.

## Conclusion

EVH for SVG harvesting did not negatively affect graft quality or patient survival. Wound-related complications such as poor healing and infection occurred at a very low rate. These results suggest that EVH is a safe and reliable technique. Flexible selection of harvesting methods based on each patient's background and comorbidities appears to be essential for optimizing surgical outcomes.

## Data Availability

The datasets presented in this study can be found in online repositories. The names of the repository/repositories and accession number(s) can be found in the article/Supplementary Material.

## References

[1] Raza S Chang C Deo SV Sabik JF 3rd. Current role of saphenous vein graft in coronary artery bypass grafting. Indian J Thorac Cardiovasc Surg. (2018) 34:245–50. 10.1007/s12055-018-0759-3 33060945 PMC7525697

[2] Samano N Souza D Dashwood MR. Saphenous veins in coronary artery bypass grafting need external support. Asian Cardiovasc Thorac Ann. (2021) 29:457–67. 10.1177/0218492320980936 33307718 PMC8167919

[3] Pu A Ding L Shin J Price J Skarsgard P Wong DR Long-term outcomes of multiple arterial coronary artery bypass grafting: a population-based study of patients in British Columbia, Canada. JAMA Cardiol. (2017) 2:1187–96. 10.1001/jamacardio.2017.3705 29049458 PMC5710366

[4] Ferrari G Loayza R Azari A Geijer H Cao Y Carlsson R Superior long-term patency of no-touch vein graft compared to conventional vein grafts in over 1500 consecutive patients. J Cardiothorac Surg. (2024) 19:570. 10.1186/s13019-024-03057-3 39354611 PMC11443723

[5] Souza DS Dashwood MR Tsui JC Filbey D Bodin L Johansson B Improved patency in vein grafts harvested with surrounding tissue: results of a randomized study using three harvesting techniques. Ann Thorac Surg. (2002) 73:1189–95. 10.1016/S0003-4975(02)03425-2 11996262

[6] Thelin S Modrau IS Duvernoy O Dalen M Dreifaldt M Ericsson A No-touch vein grafts in coronary artery bypass surgery: a registry-based randomized clinical trial. Eur Heart J. (2025) 46:1720–9. 10.1093/eurheartj/ehaf018 39969129 PMC12055231

[7] Williams JB Peterson ED Brennan JM Sedrakyan A Tavris D Alexander JH Association between endoscopic vs open vein-graft harvesting and mortality, wound complications, and cardiovascular events in patients undergoing CABG surgery. JAMA. (2012) 308:475–84. 10.1001/jama.2012.8363 22851114 PMC3699197

[8] Ouzounian M Hassan A Buth KJ MacPherson C Ali IM Hirsch GM Impact of endoscopic versus open saphenous vein harvest techniques on outcomes after coronary artery bypass grafting. Ann Thorac Surg. (2010) 89:403–8. 10.1016/j.athoracsur.2009.09.061 20103309

[9] Nakamura K Arai S Kobayashi K Nakai S Sho R Ishizawa A Safe and promising outcomes of in-hospital preoperative rehabilitation for coronary artery bypass grafting after an acute coronary syndrome. BMC Cardiovasc Disord. (2024) 24:139. 10.1186/s12872-024-03757-7 38438846 PMC10910820

[10] Nakamura K Hamasaki A Uchida T Kobayashi K Sho R Kim C The use of prophylactic intra-aortic balloon pump in high-risk patients undergoing coronary artery bypass grafting. PLoS One. (2019) 14:e0224273. 10.1371/journal.pone.0224273 31658283 PMC6816571

[11] Sohn SH Kang Y Kim JS Choi JW Hwang HY. The impact of perivascular tissue preservation on 5-year patency of saphenous vein composite grafts. Interdiscip Cardiovasc Thorac Surg. (2024) 38:ivae069. 10.1093/icvts/ivae069 38637939 PMC11076921

[12] Zivkovic I Krasic S Stankovic M Milacic P Milutinovic A Zdravkovic D Influence of three different surgical techniques on microscopic damage of saphenous vein grafts-A randomized study. Medicina. (2023) 59:217. 10.3390/medicina59020217 36837419 PMC9962261

[13] Yokoyama Y Shimamura J Takagi H Kuno T. Harvesting techniques of the saphenous vein graft for coronary artery bypass: insights from a network meta-analysis. J Card Surg. (2021) 36:4369–75. 10.1111/jocs.15974 34472140

[14] Lopes RD Hafley GE Allen KB Ferguson TB Peterson ED Harrington RA Endoscopic versus open vein-graft harvesting in coronary-artery bypass surgery. N Engl J Med. (2009) 361:235–44. 10.1056/NEJMoa0900708 19605828

[15] Zenati MA Shroyer AL Collins JF Hattler B Ota T Almassi GH Impact of endoscopic versus open saphenous vein harvest technique on late coronary artery bypass grafting patient outcomes in the ROOBY (randomized on/off bypass) trial. J Thorac Cardiovasc Surg. (2011) 141:338–44. 10.1016/j.jtcvs.2010.10.004 21130476

[16] Wang W Liu Y Qi H Liu Y Jiang Y Fan R Mid-term outcomes of endoscopic vein harvesting in coronary artery bypass grafting: a retrospective cohort study. J Cardiothorac Surg. (2024) 19:389. 10.1186/s13019-024-02930-5 38926738 PMC11210013

[17] Kodia K Patel S Weber MP Luc JGY Choi JH Maynes EJ Graft patency after open versus endoscopic saphenous vein harvest in coronary artery bypass grafting surgery: a systematic review and meta-analysis. Ann Cardiothorac Surg. (2018) 7:586–97. 10.21037/acs.2018.07.05 30505742 PMC6219959

[18] Ran J Liu Y Li Y Li Q Tang Y Deng L The effect of endoscopic vein harvesting in coronary artery bypass surgery. J Thorac Dis. (2020) 12:1991–8. 10.21037/jtd-20-250 32642102 PMC7330341

[19] Harky A Balmforth D Shipolini A Uppal R. Is endoscopic long saphenous vein harvesting equivalent to open harvesting technique in terms of graft patency? Interact Cardiovasc Thorac Surg. (2017) 25:323–6. 10.1093/icvts/ivx049 28475708

[20] Chernyavskiy A Volkov A Lavrenyuk O Terekhov I Kareva Y. Comparative results of endoscopic and open methods of vein harvesting for coronary artery bypass grafting: a prospective randomized parallel-group trial. J Cardiothorac Surg. (2015) 10:163. 10.1186/s13019-015-0353-3 26563714 PMC4642617

[21] Gaudino M Sandner S. The SWEDEGRAFT trial: when absence of evidence is not evidence of absence. Eur Heart J. (2025) 46:1730–2. 10.1093/eurheartj/ehaf043 40036740

